# Case report: Large follicular thyroid carcinoma with multiple cervical lymph node metastases

**DOI:** 10.3389/fsurg.2022.995859

**Published:** 2022-08-23

**Authors:** Fei Ye, Liyan Liao, Wanlin Tan, Yi Gong, Xiaodu Li, Chengcheng Niu

**Affiliations:** ^1^ Department of Thyroid Surgery, The Second Xiangya Hospital, Central South University, Changsha, China; ^2^ Department of Pathology, The Second Xiangya Hospital, Central South University, Changsha, China; ^3^ Department of Ultrasound Diagnosis, The Second Xiangya Hospital, Central South University, Changsha, China; ^4^ Research Center of Ultrasonography, The Second Xiangya Hospital, Central South University, Changsha, China

**Keywords:** follicular thyroid carcinoma (FTC), cervical lymph node metastases, vascular invasion, PET/CT, thyroid ultrasonography, TERT promoter mutation

## Abstract

**Introduction:**

Follicular thyroid carcinoma (FTC) rarely metastasizes to regional lymph nodes, as they mainly metastasize through hematogenous route; in particular, a large FTC with only lateral lymph node metastasis and without distant metastasis has rarely been reported.

**Case report:**

We present a 66-year-old male patient with a progressively growing thyroid for more than 20 years, causing tracheal compression and narrowing. Neck ultrasonography, computed tomography (CT), magnetic resonance (MR) imaging and positron emission tomography–computed tomography (PET/CT) were carried out to obtain images of the thyroid and surrounding tissues. Total thyroidectomy and cervical lateral and central lymph node dissection were undertaken, and histopathological, and immunohistochemical evaluations and molecular pathology confirmed the diagnosis of FTC with multiple cervical lymph node metastases.

**Conclusion:**

We have reported a rare case of large FTC with diffuse nodal involvement but no distant metastases. We present the thyroid ultrasound, neck CT, MR and whole body PET/CT.

## Introduction

Follicular thyroid carcinoma (FTC) is a type of differentiated thyroid cancer (DTC) derived from follicular cells that accounts for 10%–15% of all thyroid cancers. Its incidence is second only to papillary thyroid carcinoma (PTC), but its mortality rate is higher than that of PTC. Some patients have bone or lung metastases during initial treatment (1, 2). FTC mainly metastasizes through the blood circulation, and usually less than 10% metastasize through lymphatic system (3, 4).

Early diagnosis and treatment and the close follow-up of patients are the main means to improve the prognosis and prolong the postoperative survival of patients with FTC. However, unlike PTC, FTC is often associated with relatively benign ultrasound features on ultrasound images and can be easily diagnosed as thyroid follicular adenoma (FA) instead, which makes clinical treatment problematic (5). Additionally, it is difficult to obtain a diagnosis from a fine needle aspiration, and the identification of cytological markers are not developed enough currently (6). FTC is often diagnosed based on clinicopathological vascular and capsular infiltration, and is sometimes missed due to a lack of clear infiltration foci or insufficient sampling, thus the diagnosis relies on morphology combined with immunohistochemical staining and molecular pathology (7).

Here, we report on a patient with a large FTC with lateral lymph node metastasis and no distant metastasis, showing the thyroid ultrasound, CT, MR and PET/CT images of the FTC and cervical lymph nodes, which provided multimodal preoperative imaging information for FTC.

## Case report

**Figure 1:**
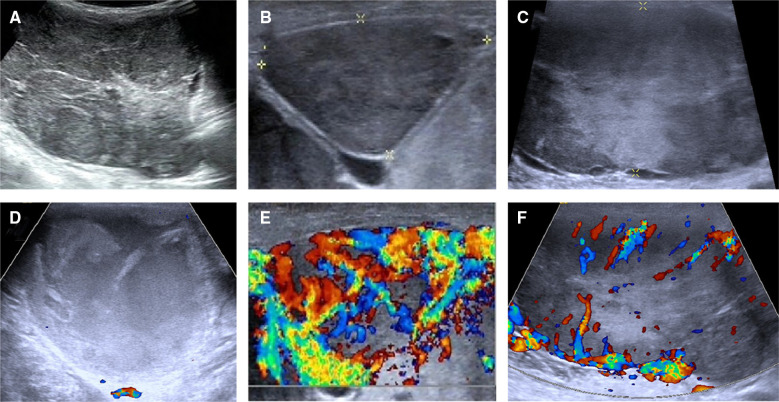
Ultrasonographic images of the thyroid and lateral lymph nodes. (**A**) The thyroid tissue displayed a heterogeneous echoic appearance on gray-scale sonography. (**B,C**) Cervical lymph nodes showed round shape and absence of hilum on gray-scale sonography. (**D–F**) CDFI showed different vascular distribution of cervical lymph nodes. (**D**) A mixed-echoic cervical lymph node with more than 90% of cystic change revealed no blood flow signal inside this lymph node. (**E**) A heterogeneous echoic cervical lymph node showed rich blood flow signal inside and around this lymph node. (**F**) A heterogeneous echoic cervical lymph node showed rich blood flow signal around this lymph node.

**Figure 2:**
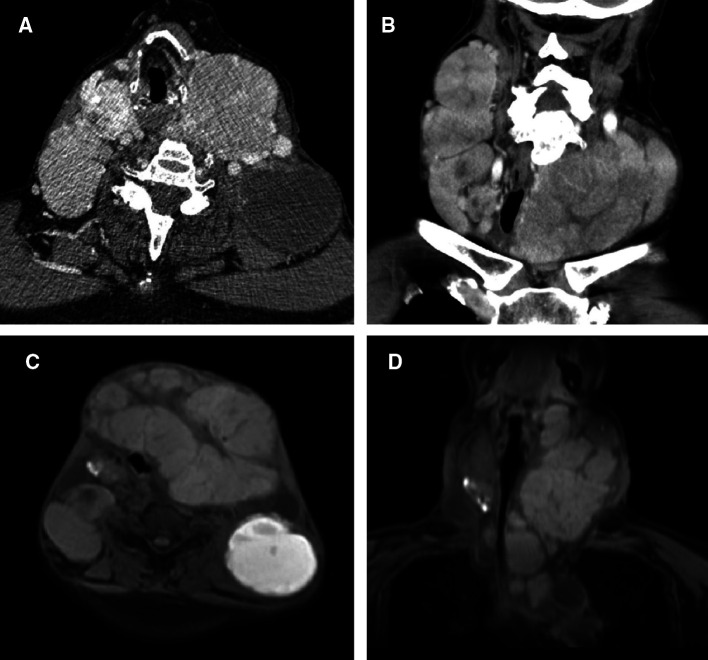
CT and MR images of the neck. (**A**) Transverse and (**B**) coronal sections of the neck revealed swelled thyroid with multiple enlarged cervical lymph nodes on CT. (**C**) Transverse and (**D**) coronal sections of the neck revealed swelled thyroid with multiple enlarged cervical lymph nodes on MR, causing the tracheal compression and narrowing.

**Figure 3:**
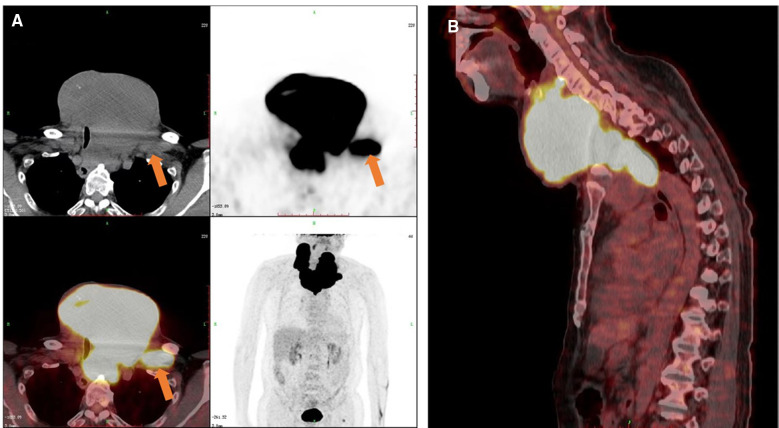
PET/CT images of the patient. Increased 18F-FDG metabolism showed in the thyroid and cervical lymph nodes on the (**A**) transverse, coronal and (**B**) sagittal sections. The orange arrow indicated one of the cervical metastatic lymph nodes.

This study was reported in agreement with principles of the CARE guidelines (8). A 66-year-old male patient presented with a progressively growing thyroid for more than 20 years, causing tracheal compression and narrowing. The neck of the patient was abnormally swollen, with engorged superficial veins. The neck ultrasound revealed diffuse and swollen heterogeneous hypo-echoic thyroid tissue (Figure 1A). Cervical lymph nodes were enlarged, round and had no hilum on grayscale sonography, with different vascular distributions on color Doppler flow imaging (CDFI) mode (Figures 1B–F). Combined with the sonographic appearance of lateral lymph nodes, the whole thyroid was highly suspicious for malignancy. The neck CT was carried out to evaluate the thyroid mass with surrounding tissues, which revealed an extremely swollen thyroid with multiple enlarged cervical lymph nodes, some of which were larger than 6 cm, and some of which had obvious cystic changes and necrosis (Figures 2A,B). Then, the neck MR was used to evaluate the degree of stenosis of the cervical trachea and the need for a tracheal stent. MR images showed uneven signal values in the enlarged thyroid and lateral lymph nodes, and the tracheal compression and narrowing was obviously observed (Figures 2C,D). The patient had a tracheal stent placed before surgery to prevent airway stenosis. Next, PET/CT with 18F-fluorodeoxyglucose (18F-FDG) was performed to assess the extent of disease in the whole body due to the large mass, which showed extensive hypermetabolic lesions in the front of the neck and partial extension to the retrosternal space (Figure 3). Fortunately, this patient had no distant metastases. In addition, the serum thyroglobulin (TG) was more than 10,000 ng/ml (normal range was 3.5–77.0 ng/ml), which was far more than the normal value. The serum anti-TG value was 1.620 IU/ml (normal range was 0.000–4.110 IU/ml).

**Figure 4:**
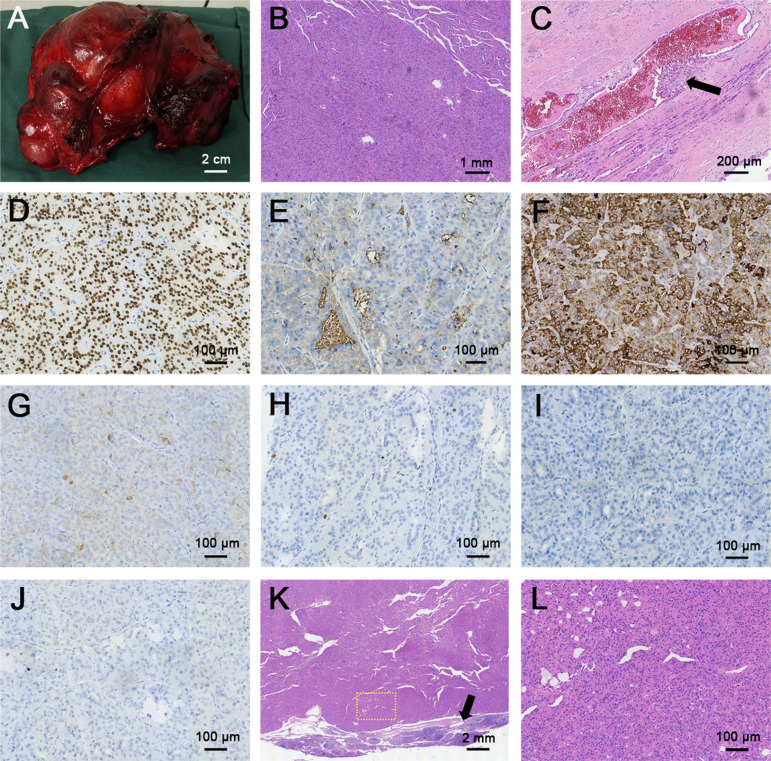
Gross and histopathological sections of follicular thyroid carcinoma and metastatic lymph node. (**A**) Gross image of follicular thyroid carcinoma. (**B,C**) H&E staining of follicular thyroid carcinoma (**B**, magnification × 40), black arrow indicated the vascular invasion of follicular thyroid carcinoma, the thyroid follicular epithelial cells were invaded into the blood vessel (**C**, magnification × 100). (**D–J**) Immunohistochemical (IHC) staining of follicular thyroid carcinoma (magnification × 200) for (**D**) TTF-1, (**E**) TG, (**F**) CK 7, (**G**) CK pan, (**H**) Ki 67, (**I**) calcitonin, (**J**) PTH. TTF-1 and CK7 were deeply stained (positive), TG and CK pan were partially stained (partial positive), Ki67 proliferation index was less than 1%, calcitonin and PTH didn't stain (negative). (**K,L**) H&E staining of neck metastatic lymph node: (**K**) magnification × 16, (**L**) magnification × 400. Yellow dashed circle indicated the amplification part in the picture **K**, black arrow indicated the normal part of the metastatic lymph node.

Then, a total thyroidectomy and cervical lateral and central lymph node dissection were undertaken; this whole thyroid was completely resected and weighted 623.2 g, which was almost 30-fold higher than normal thyroid weight (Figure 4A). The pathology report revealed a large diffuse infiltrative FTC with the longest diameter of 16.0 cm and more than 4 vascular invasion lesions (tumor cells invading a vessel wall indicates with a black arrow, Figure 4C), and nine cervical lymph nodes were removed and all of them showed metastatic involvement of FTC (Figures 4K,L). According to the eighth edition of the American Joint Committee on Cancer/Tumor Lymph Node Metastasis (TNM) staging system, the patient was in TNM stage II (Any T, N1, M0) (9). The immunohistochemical stains of FTC were positive for thyroid transcription factor (TTF-1) and cytokeratin (CK) 7, partially positive for thyroglobulin (TG) and CK pan, and negative for calcitonin and parathyroid hormone (PTH), and the Ki67 proliferation index was less than 1%, which is consistent with the diagnosis of angio-invasive FTC (Figures 4D–J). Next-generation sequencing (NGS) results revealed epidermal growth factor receptor (EGFR) and telomerase reverse transcriptase (TERT) promoter region (c.-124C > T) mutations with tumor mutational burden (TMB) of 2.13 mutations/Mb.

The patient did not undergo ablative radioiodine therapy post surgery due to non-medical reasons. The TG concentration was measured 8 months after surgery and it was 300 ng/ml (the normal value after surgery should not exceed 1 ng/ml). The anti-TG was less than 1.980 IU/ml (normal range was 0.000–4.110 IU/ml), while TSH was more than 54.744 mU/L (normal range was 0.51–4.91 mU/L) because the patient reduced the doses of L-thyroxin by personal reason last 2 weeks. Enlarged bilateral cervical lymph nodes were found in neck on ultrasonography and isotope I-131 whole-body scan and SPECT that required second surgery followed by radioiodine therapy. Subsequently, the patient will be treated with suppressive doses of L-thyroxin. Fortunately, the patient has completed relevant examinations and is about to receive radioiodine therapy recently.

## Discussion

FTC accounts for approximately 10% of the incidence of thyroid cancer and is prone to distant metastasis *via* blood vessels; in addition, FTC is more aggressive than PTC, develops distant metastases more frequently and shows higher mortality rate (2). Patient with FTC often experience no obvious clinical symptoms; therefore, FTC is mostly found when an ultrasound examination finds a single nodule, or when the patient is admitted to hospital due to related clinical symptoms associated with distant metastases. The most common sites of metastases are the lungs and bones, followed by the brain, liver and skin (3). In this case, this patient had a history of swollen thyroid for many years, but he had not received any treatment because there had been no obvious pressure symptoms of dyspnoea, dysphonia or dysphagia. He presented to our hospital with a progressively enlarged thyroid accompanied by tracheal compression and narrowing. Fortunately, this large FTC only had cervical metastatic lymph node involvement without distant metastases. However, lymph node metastases are rare in patients with FTC and usually have an average incidence of less than 10%; though, in this specific case, there were only lymph node metastases and no signs of haematogenic spread.

Thyroid ultrasonography is the most commonly used examination to evaluate thyroid nodules, and given that FTC and FA have similar appearances in color ultrasonography, and they may be misdiagnosed by inexperienced sonographers. Typical FTC is mainly characterized by an ill-defined shape, no fine halo, inhomogeneous echogenicity, coarse calcification and solid composition, while FA often shows a clear border, regular shape, fine halos, absence of calcification and solid composition (5, 10). In this case, thyroid ultrasonography showed a diffuse heterogeneous echoic appearance of whole thyroid tissue, which was not consistent with the sonographic findings of nodular FTC, while the postoperative pathology was confirmed as diffuse FTC. Ultrasonography is also the recommended method to evaluate benign and malignant cervical lymph nodes. In this case, all cervical lymph nodes were round and lacked a hilum, and some of them showed obvious cystic changes on grayscale sonography. Most of the lymph nodes displayed rich central and peripheral vascularity in the lymph node, the others showed peripheral vascularity, while some had no obvious vascularity when the lymph nodes had more than 90% cystic change. The ultrasonographic features of the lymph nodes were consistent with the reported sonographic findings of metastatic lymph nodes, and the postoperative pathology confirmed metastatic lymph nodes from FTC.

CT, MRI and PET/CT examinations are also important for accurate preoperative assessment (11). FTC is mainly metastasized *via* the blood circulation, and once the primary thyroid tumor is suspected to be FTC, patients with suspected FTC in other organs should undergo CT, MRI, PET/CT and other related examinations for a comprehensive evaluation. Parghane et al. reported on a 54-year-old male FTC patient presenting with cough and occasional hemoptysis for 1 year and hematuria for 6 months (12). A CT scan showed heterogeneously enhancing soft tissue lesions in the hilar region of the right lung, right lateral wall of the urinary bladder, segment III of the liver, and destruction of the left 5th rib. A PET/CT showed intense 18F-FDG metabolism in multiple mediastinal lymph node lesions, three liver lesions, and multiple left thoracic and skeletal lesions. Therefore, FTC patients under initial treatment or those with recurrent metastasis following surgical treatment or various other local or systemic treatments, should be evaluated for their efficacy to guide the formulation of further diagnosis and treatment or follow-up strategies.

The histopathological diagnosis of FTC requires the determination of capsular and/or vascular invasion and can be divided into the following three categories: microinvasive, encapsulated vascular invasion, and diffuse invasion. The microinvasion of local blood vessels or capsules usually involves less than 4 blood vessels or capsule infiltration. Diffuse invasion usually has a poor prognosis, a high risk of recurrence and metastasis, and easily metastasizes to bone, lung, and occasionally to the soft tissue, liver or brain. In DTC, the mutation rate of RAS is second only to BRAF, and RAS mutation is the most common in FTC (13). However, RAS gene mutations can occur in both malignant and benign thyroid nodules, and the use of RAS alone in the differential diagnosis of benign and malignant thyroid nodules and the assessment of prognosis has limitations. Recent studies have found that TERT promoter mutation is closely related to high tumor aggressiveness, and TERT promoter mutated FTCs may exhibit a specific miRNA pattern that in part regulates key cancer pathways (14–16). Detection of the BRAF, RAS and TERT promoters is helpful for evaluating and predicting the biological behavior of thyroid cancer (17, 18). In this case, the patient had the TERT promoter mutation, but exhibited only cervical lymph node metastases and no distant metastases, which was rarely reported. Unfortunately, the current mechanism in this case is not very clear.

Surgery is the main treatment modality for FTC. In the ATA guidelines, there is no difference between the surgical recommendations for PTC and FTC (11). Post-surgery ablative radioiodine treatment is recommended in all FTC cases, not only the aggressive cases. Furthermore, an adequate TSH suppression prolongs survival in high-risk thyroid cancer patients (19). For FTC patients, the presence or absence of distant metastasis directly affects the survival rate. Patient age, vascular and capsular invasion, and surgical methods are all related to tumor recurrence, metastasis and prognosis (20). In this case, the patient underwent total thyroidectomy and cervical lymph node dissection. However, the patient had recurrent lymph node metastases and extremely high TG level, which required second surgery followed by radioiodine therapy.

According to ATA guidelines, this patient was classified in ATA high risk. The patient needs to receive subsequent radioiodine therapy, and the TSH levels should be suppressed with L-thyroxin to <0.1 mU/L (11).

Unfortunately, the post-surgical management of this patient was not in line with the ATA guidelines. The patient did not receive ablative radioiodine treatment for non-medical reasons. The lack of appropriate ablation has led to early recurrence in the cervical lymph nodes, after 8 months the patient had structural (enlarged bilateral cervical lymph nodes in neck on Ultrasonography and isotope I-131 labeled SPECT of the neck region) and functional (serum Tg > 300 ng/ml) evidence of loco-regional metastases, which required second surgery followed by radioiodine therapy. Subsequently, the patient will be treated with suppressive doses of L-thyroxin.

FTC is sometimes missed due to a lack of clear infiltration or to insufficient sampling; it is difficult to use ultrasonography and FNA for diagnosis; and cytological marker detection is currently not sufficiently developed. Therefore, we need to raise awareness of FTC.

## Conclusion

In this paper we have reported on a rare case of large FTC with diffuse nodal involvement but no distant metastases. We present the thyroid ultrasound, neck CT, MR and whole body PET/CT.

## Data Availability

The original contributions presented in the study are included in the article/Supplementary Material, further inquiries can be directed to the corresponding author/s.
